# Dravet Syndrome Associated With a CSNK2B-Related Neurodevelopmental Disorder

**DOI:** 10.7759/cureus.104138

**Published:** 2026-02-23

**Authors:** Abena O Yeboah, Charles D Tyshkov, Suman Ghosh

**Affiliations:** 1 Pediatrics, Maimonides Medical Center, New York City, USA; 2 Pediatrics, Weill Cornell Medicine, New York City, USA; 3 Neurology, Maimonides Medical Center, New York City, USA; 4 Neurology, State University of New York (SUNY) Downstate College of Medicine, New York City, USA

**Keywords:** developmental and epileptic encephalopathies, intractable epilepsy, severe myoclonic epilepsy in infancy, synapse, synaptic

## Abstract

Dravet syndrome (DS) is a developmental epileptic encephalopathy characterized by prolonged febrile and afebrile focal clonic as well as generalized seizures. The most common genetic etiology is SCN1A, but other genetic disorders can present with the phenotype. A two-year-and-11-month-old Caucasian female patient of Russian descent, with a history of developmental delay and recurrent prolonged febrile focal clonic seizures since the age of 2-3 months, presented with afebrile status epilepticus. Genetic testing identified a de novo mutation in CSNK2B. Her clinical history and semiology were consistent with DS. The CSNK2B mutation is associated with neurodevelopmental disorders characterized by developmental delays and epilepsy of varying severity. This is the first reported case of a child with a CSNK2B mutation presenting with a DS phenotype. The pathophysiological mechanisms of CSNK2B mutations involve disrupted casein kinase 2 (CK2) activity, which impacts synaptic plasticity and shares similarities with the synaptic dysfunction seen in other genes associated with the DS phenotype. Identifying such cases expands the knowledge base and understanding of the genetic landscape of DS.

## Introduction

Dravet syndrome (DS) is a developmental epileptic encephalopathy with an incidence of one in 15,700 in the United States [1]. DS presents in the first year of life in a normally appearing child with recurring prolonged febrile and afebrile seizures, which are often focal clonic (hemiclonic) or generalized clonic. Other types of seizures, such as myoclonic, atonic, focal awareness impaired, and absence seizures, may appear during the first four years of life. The diagnosis of DS is frequently delayed until clinical features converge due to the severity of the condition. The initial seizure is typically a prolonged hemiclonic seizure associated with fever, elevated body temperature, or vaccination. Variability in onset is noted, with 28%-61% of patients experiencing febrile seizures frequently linked to vaccination or illness [2,3]. These seizures can evolve into prolonged episodes lasting over 20 minutes, leading to status epilepticus. Early EEGs are typically normal except for postictal slowing. Initially mistaken for febrile seizures, the diagnosis becomes apparent between ages one and four years, characterized by multiple seizure types, early pharmacoresistance, and significant developmental delays [4]. Comorbidities in DS include motor delays, language delays, difficulty with coping or personal skills, sleep disorders, and learning disabilities [1]. Mortality occurs in 3.7-20.8% of patients with DS, which is significantly higher than age- and sex-matched controls [5]. DS negatively affects quality of life (physical, psychosocial, emotional, and daily function) for both the child and the parents. Parents caring for children with DS report higher symptoms of depression and financial concerns due to the cost of care, hospitalizations, and treatment [5].

The diagnosis is based on clinical history, with over 80% of patients possessing a de novo pathogenic mutation of the SCN1A gene variant, which encodes for the alpha subunit of the voltage-gated sodium channel [2]. Additional genes have also been identified in patients with DS, including PCDH19, GABRA1, STXBP1, CHD2, SCN1B, SCN2A, KCNA2, HCN1, and GABRG2. DNA analysis conducted on venous blood samples of 36 patients with DS-like phenotypes showed 15 of these patients (41.7%) had SCN1A mutations, one patient (2.8%) had an SCN8A mutation, one patient (2.8%) had an STX1B mutation, five female patients (13.9%) had PCDH19 mutations, and 14 patients (38.9%) had an unknown diagnosis or genetic mutation [3]. Early identification of patients with DS may result in early treatment with appropriate antiseizure medications such as cannabidiol, fenfluramine, and stiripentol, which appear to have better efficacy [6]. Part of the challenge in diagnosis lies in novel gene mutations that may present with the phenotype but may not be classified as DS since they do not carry the most common mutation. We present a previously identified patient with CSNK2B-related neurodevelopmental disorder (CSNK2B-NDD) who fits the syndromic diagnosis of DS.

## Case presentation

A two-year-and-11-month-old female patient with a history of seizures on levetiracetam presented to Maimonides Hospital in September 2024 with a chief complaint of recurrent seizures throughout the day. The patient was reportedly at her baseline when she was noted to have tactile fever, after which she developed two episodes of generalized tonic-clonic seizures lasting approximately one minute each. She returned to baseline and then developed two more episodes of general tonic-clonic seizure with postictal vomiting of similar duration, after which she was brought to the emergency department. 

She appeared stuporous on exam when not seizing. The patient was afebrile and tachycardic with no tachypnea on room air. She had no dysmorphic features and no cranial nerve deficits, was hypotonic but moved extremities antigravity to stimuli, and exhibited intact deep tendon reflexes and a bilateral extensor plantar response. She remained in status epilepticus despite receiving lorazepam 0.1 mg/kg and levetiracetam 55 mg/kg bolus. She was transferred to the pediatric intensive care unit (PICU) to initiate a midazolam IV infusion, after which her seizures stopped.

**Figure 1:**
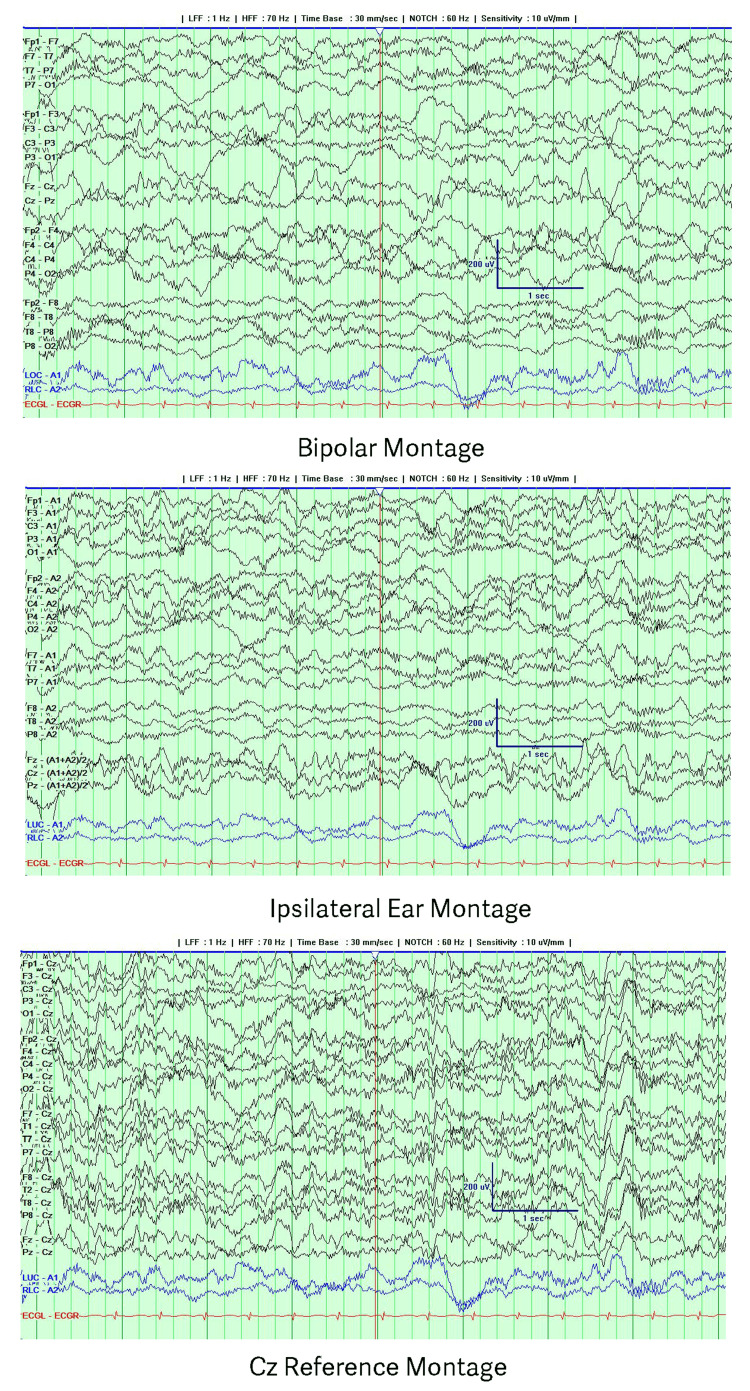
EEG during intubation while on midazolam showing generalized delta slowing and excessive beta The recording is presented using a bipolar montage with ipsilateral ear and Cz references.

The exam was nonfocal except for diffuse hypotonia and a bilateral extensor plantar response while lowering sedation. A comprehensive electrolyte panel, serum inflammatory markers, and complete blood count were unremarkable. Blood and urine cultures remained negative after five days. The urine toxicology screen was negative. A head CT was unremarkable. Video EEG performed while intubated on midazolam showed continuous generalized delta slowing with excessive beta (Figure 1).

**Figure 2:**
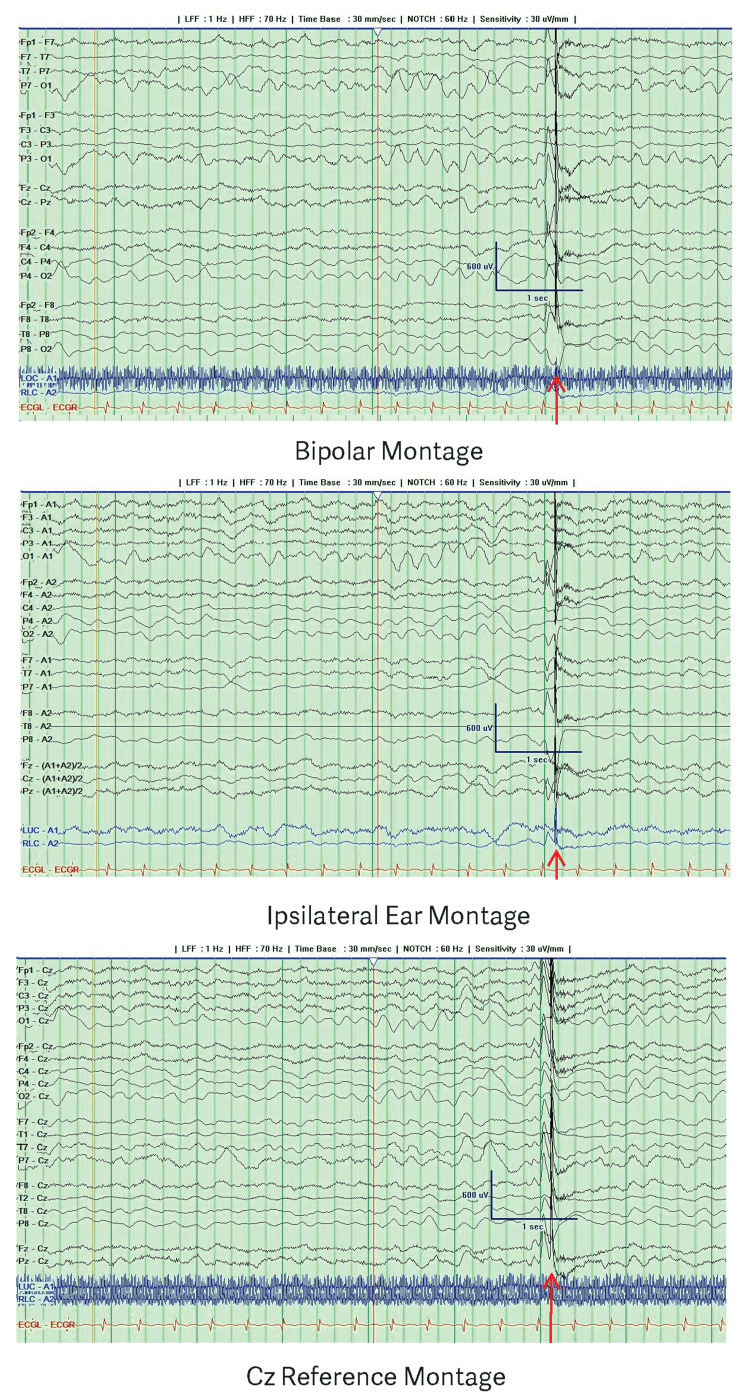
EEG two hours after extubation and discontinuation of midazolam showing a posterior dominant rhythm of 5 Hz and an interictal generalized spike-wave complex The red arrow indicates a generalized spike-wave complex. The recording is presented using a bipolar montage with ipsilateral ear and Cz references.

Video EEG after extubation and cessation of midazolam for two hours showed a posterior dominant rhythm of 5 Hz and an interictal generalized spike-wave complex (Figure 2).

Per history from the parents and her primary neurologist, she was a full-term infant delivered vaginally with adequate prenatal care in Russia. Her first seizure began between two and three months of age in Russia, following a fever possibly triggered by her two-month vaccinations. These initial seizures involved one side of her body. After her first seizure, she was started on levetiracetam and then transitioned to valproate in Russia. Her parents recalled that the child was able to roll and sit but had only recently gained the ability to walk a few months prior to admission at two years and 11 months of age. Speech remained limited to one-word responses and nonsensical babbling. Before her admission to Maimonides for status epilepticus, she had experienced a total of 12 febrile seizures. There was no family history of developmental delay, autism, epilepsy, or other neurological disorders. An EEG performed in Russia had reportedly been normal, but a subsequent EEG report from an outside hospital in September 2022 revealed occasional independent interictal epileptiform discharges in the right frontocentral and left frontal regions. She was transitioned back to levetiracetam, and valproate was discontinued. In February 2023, genetic testing confirmed a de novo autosomal dominant mutation in the CSNK2B gene. At follow-up in September 2023, the patient continued to show significant developmental delays with poor language acquisition and delays in ambulation.

Based on her clinical history and seizure semiology, she was diagnosed with DS in September 2024. Other diagnoses in the differential at the time included Dravet-like syndrome and Lennox-Gastaut syndrome, although she did not have diffuse generalized slow spike and wave on EEG. She was discharged home on clobazam 5 mg twice a day and levetiracetam 55 mg/kg/day with neurological follow-up. She subsequently returned multiple times to the Maimonides emergency room for recurring seizures. By the age of four years, she had sustained 32 seizures despite being on maximal dosing of levetiracetam and clobazam. Given her current refractory state, there is active discussion about starting a third antiseizure medication. 

## Discussion

The patient’s phenotype is consistent with DS or Dravet-like syndrome. Autosomal dominant mutations in the CSNK2B gene are associated with a wide range of neurodevelopmental disabilities and varying degrees of epilepsy severity [7]. A literature search in PubMed, Web of Science, Clinvar, and Online Mendelian Inheritance in Man (OMIM) databases revealed no documented association between this mutation and DS or the DS phenotype. This patient presentation with a DS phenotype has not been previously reported in the literature. 

CSNK2B-NDD is characterized in most reported individuals - approximately 80-89% - by developmental delay or intellectual disability and seizure disorder [7,8]. Young children commonly present with delays in speech and motor development. Among individuals older than five years at the time of evaluation, intellectual disability ranges from borderline or mild to severe or profound. Seizures, observed in the majority of patients, vary in type and severity [8]. While many individuals have pharmacoresponsive epilepsy, others experience severe epilepsy with recurrent episodes of refractory status epilepticus. Less consistent findings include ataxia, hypotonia in infancy, neurobehavioral manifestations, and musculoskeletal anomalies.

CSNK2B-NDD is caused by a mutation in casein kinase 2 (CK2), a serine/threonine kinase composed of catalytic and regulatory subunits that regulates key cellular processes, including survival, proliferation, differentiation, and synaptic plasticity, particularly in the brain. Both CK2 and voltage-gated sodium channels play critical roles in synaptic transmission, synaptic potentiation, and neuronal function [9]. CK2 activity increases during long-term potentiation, and CK2-selective inhibitors block NMDA-receptor-dependent synaptic transmission and long-term potentiation [9,10]. Voltage-gated sodium channels are essential for initiating action potentials and maintaining neuronal excitability. Loss-of-function mutations, particularly in inhibitory interneurons, have been linked to conditions like DS, where disrupted inhibition leads to excessive neuronal excitation and seizures [11]. Both genes are associated with autosomal dominant disorders that impact synaptic function, leading to neuronal excitability.

## Conclusions

DS encompasses several gene mutations related to synaptic transmission and neuronal excitability. This common feature may facilitate further research in identifying broader therapeutic modalities targeting synaptic transmission and neuronal excitability for developmental epileptic encephalopathies. Recognition of CSNK2B-NDD as a cause of DS can help patients obtain optimal seizure management and enroll in antiseizure drug trials for children with seizures from this syndrome.
